# Complete Genome Sequence of a Bovine Viral Diarrhea Virus Subgenotype 1e Strain Isolated in Switzerland

**DOI:** 10.1128/genomeA.00636-15

**Published:** 2015-06-11

**Authors:** Hanspeter Stalder, Matthias Schweizer, Claudia Bachofen

**Affiliations:** a Institute of Virology and Immunology, Federal Food Safety and Veterinary Office FSVO and Vetsuisse Faculty University of Bern, Bern, Switzerland; b Institute of Virology, Vetsuisse Faculty University of Zurich, Zurich, Switzerland

## Abstract

We sequenced the complete genome of the bovine viral diarrhea virus (BVDV) strain Carlito. It belongs to the subgenotype 1e that is described in Europe only and represents the second most prevalent subgenotype in Switzerland. This is the first report of a full-length sequence of BVDV-1e.

## GENOME ANNOUNCEMENT

Bovine viral diarrhea virus (BVDV) is an important pathogen of ruminants throughout the world (1), causing severe economic losses to the cattle industry (2). The two species BVDV-1 and BVDV-2 are part of the genus *Pestivirus* in the family *Flaviviridae*. BVDV-1 is further subdivided into 11 to 16 subgenotypes (3, 4). Several European countries have ongoing BVDV eradications (for review, see references 1, 5, and 6). In the course of the Swiss eradication program, BVDV-1e was isolated from the blood of the Red Holstein calf Carlito in 2012. BVDV-1e has only been described in Europe. It seems to be the predominant BVDV-1 subgenotype in France (7) but has also been reported in Austria, Czech Republic, Denmark, Italy, Portugal, Slovakia, and Spain (3, 8–14). In Switzerland, BVDV-1e is the second most prevalent subgenotype; around one-third of all isolates sequenced belong to this subgenotype (15, 16).

Here, we describe the first full-length sequence of a BVDV-1e strain. Virus from blood was isolated on MDBK cells. Total RNA was extracted from the cell culture supernatant using TRIzol LS (LifeTechnologies). Sequence-independent single primer amplification (SISPA) was used for reverse transcription and nonspecific amplification (17). Sequencing libraries were constructed using the IonXpress Plus library preparation kit (LifeTechnologies) and subjected to next generation sequencing using the IonTorrent PGM sequencer at the Functional Genomic Center, Zurich. Alignment of the trimmed reads to full-length BVDV sequences from GenBank allowed the determination of about 80% of the Carlito genome in 3 contigs. Gaps between the contigs were filled by direct DNA-Sanger cycle sequencing that was performed by Microsynth (Balgach, Switzerland) with BigDye Terminator chemistry (v3.1) and capillary electrophoresis (ABI 3730xl DNA analyzer; Applied Biosystems). The 5′ and 3′ ends were determined by a simplified protocol for rapid amplification of cDNA ends (RACE) (18), omitting the cloning step but performing direct sequencing of the amplification products. The electropherograms obtained were assembled with the SeqMan II sequence analysis software (v5.01; DNAStar, Inc., Madison, WI) and the sequences analyzed using the Clone Manager 9 Professional Edition (Scientific & Educational Software, Cary, NC) and the MEGA program v5.05 (19).

The complete genome of the strain Carlito comprises 12,264 nucleotides (nt), with 5′ and 3′ untranslated regions (UTRs) of 383 nt and 184 nt, respectively. The single large open reading frame codes for 3,898 amino acids. Compared to other subgenotypes, 1e is rather heterogeneous, displaying also significant antigenic differences to other BVDV-1 subgenotypes (4, 16). The virus shares only 74% to 81% nucleotide homology with other published full-length BVDV-1 genomes. For the 5′ UTR and *N*^pro^ regions, sequences of all BVDV-1 subgenotypes have been published. In contrast, only very limited data is available on full-length BVDV-1 isolates other than 1a and 1b, which are the most prevalent subgenotypes in the United States and United Kingdom. Therefore, the publication of the first full-length BVDV-1e sequence, a frequent subgenotype in several European countries, represents an important contribution to filling this gap.

### Nucleotide sequence accession number.

The genomic sequence of strain Carlito has been deposited in GenBank under the accession no. KP313732.
